# Supplementary material for: Survey of clot waveform analysis of normal activated partial thromboplastin time in patients with cirrhosis and sepsis at Le Van Thinh hospital
Source: PLoS One. 2025 Oct 24;20(10):e0334920. doi: 10.1371/journal.pone.0334920 (PMC12551873; doi:10.1371/journal.pone.0334920)
Supplement: S1 File — (PDF) [file pone.0334920.s001.pdf]

aPTT	aPTT (%)	Max1	Max2	Min2	delOD
26.6	0.87	382.452	1050.977	328.973	536.122
32.8	1.07	407.087	991.887	398.638	521.32
29.3	0.98	361.822	1009.999	312.797	449.109
26.4	0.88	506.116	1743.252	366.093	671.581
36.3	1.21	518.452	1352.453	465.134	673.68
33.5	1.12	594.786	1247.803	259.216	1583.15
33	1.1	242.188	765.841	223.836	266.936
29.9	1	423.73	1396.037	369.956	519.868
33.1	1.1	313.172	835.967	340.431	361.524
30.4	1.01	141.879	293.793	89.515	232.133
28	0.91	849.882	2065.303	808.935	1091.399
28.1	0.94	392.386	1186.05	492.543	381.578
35	1.17	333.094	865.632	388.897	361.692
31.7	1.06	453.294	1048.999	312.808	705.508
36.2	1.18	299.059	700.976	251.811	444.257
35.8	1.17	281.012	721.397	236.528	417.164
32.6	1.06	195.077	607.409	182.349	219.963
32.2	1.05	592.056	1246.139	393.754	1068.518
28.7	0.93	307.857	914.893	378.873	315.875
31.2	1.02	401.949	1047.862	476.743	431.206
27.1	0.88	338.479	904.294	273.706	480.716
32.9	1.07	331.483	716.38	274.221	472.087
31.2	1.04	348.561	1194.189	229.404	483.85
30.2	1.01	416.469	1235.223	240.058	778.921
36.2	1.21	174.416	507.879	103.975	301.445
32.8	1.09	425.193	1312.859	377.571	461.731
31.7	1.06	808.405	2310.245	594.636	1143.435
31.2	1.04	317.241	997.299	290.641	369.197
33.6	1.12	126.559	409.546	108.516	146.544
34.7	1.16	467.159	1155.943	462.363	606.628
36.7	1.22	483.342	1447.905	321.9	814.474
32.3	1.05	206.376	573.102	135.048	313.629
35	1.14	565.907	1386.287	563.558	687.028
29.9	0.97	318.942	923.965	356.414	355.562
33.5	1.09	111.562	359.353	119.016	154.599
28.2	0.92	214.184	772.255	180.441	229.927
31.8	1.04	248.273	690.205	248.788	298.947
30.6	1	298.364	915.477	327.601	309.423
30.4	0.99	261.813	835.24	291.557	276.498
28.1	0.92	170.105	580.645	253.377	135.413
27.5	0.9	263.79	869.109	291.173	275.237
33.4	1.09	702.818	1398.77	590.747	1029.445
33.1	1.08	212.563	696.387	256.623	191.98
27.4	0.89	511.718	1634.912	694.767	457.711
36.2	1.18	292.751	902.428	331.837	304.95
35.4	1.15	137.779	413.096	124.074	166.582
36.2	1.18	412.589	1039.147	476.553	454.184
32.9	1.1	115.573	407.06	166.358	110.808

30.2	1.01	248.707	693.637	334.469	226.614
33.7	1.12	535.7	1325.922	496.612	672.03
28.8	0.94	146.397	463.839	138.995	166.764
33.1	1.08	329.934	1084.183	290.612	375.536
30.9	1.01	279.88	761.991	305.073	332.837
33.9	1.13	406.774	1321.183	314.154	503.022
29.5	0.96	224.259	633.059	287.85	231.878
34.4	1.15	307.902	882.042	417.765	292.294
26	0.85	248.286	740.402	339.945	230.323
33.8	1.1	236.494	558.1	261.591	283.579
27.7	0.9	544.843	985.366	592.374	651.511
30.3	0.99	283.358	865.01	350.981	279.742
28.7	0.93	337.48	1031.307	460.808	316.051
31.5	1.03	230.662	722.17	312.464	208.666
31.5	1.03	370.558	917.167	362.996	484.645
33.1	1.08	243.385	657.394	285.398	269.289
27.5	0.9	224.547	693.38	332.761	198.156
27.2	0.89	232.754	761.265	297.156	207.884
29.1	0.95	256.367	703.792	374.278	241.276
29.3	0.98	296.141	951.179	360.229	300.246
29.5	0.98	188.307	597.589	290.785	145.232
26.4	0.88	257.281	878.048	347.8	239.366
32.9	1.07	238.336	594.137	305.565	241.504
33.8	1.1	282.262	658.151	322.317	326.209
33.4	1.09	257.016	761.573	330.333	225.651
28.7	0.96	222.462	641.753	199.119	265.167
27.7	0.9	337.244	991.794	401.292	352.216
34	1.11	216.285	640.519	223.049	218.273
28.7	0.93	216.934	739.009	201.211	228.463
30.1	0.98	295.139	908.929	294.762	343.307
29	0.97	361.802	1110.85	561.255	298.316
27.5	0.9	368.229	953.763	415.402	421.31
35.7	1.16	337.741	722.137	386.756	389.348
33.5	1.12	226.97	691.49	285.098	210.942
32.4	1.08	265.239	686.452	306.556	303.321
32.4	1.08	357.583	895.965	458.423	360.746
29	0.97	299.362	957.451	413.032	260.666
30.8	1.03	275.28	851.509	387.685	247.697
28.7	0.96	299.876	940.212	349.491	329.203
34	1.13	292.727	836.882	427.158	254.85
27.9	0.93	257.49	803.717	357.809	255.481
34.1	1.14	198.757	486.857	209.545	253.735
36.7	1.2	260.441	654.423	330.903	281.046
33.8	1.1	291.704	681.992	277.902	399.138
30.1	0.98	295.139	908.929	294.762	343.307
32.5	1.06	307.204	876.27	406.177	301.146
36.5	1.19	258.079	567.634	256.451	340.01
