# Supplementary material for: Survey of clot waveform analysis of normal activated partial thromboplastin time in patients with cirrhosis and sepsis at Le Van Thinh hospital
Source: PLoS One. 2025 Oct 24;20(10):e0334920. doi: 10.1371/journal.pone.0334920 (PMC12551873; doi:10.1371/journal.pone.0334920)
Supplement: Attachment — Submitted filename: Response to reviewers.pdf [file pone.0334920.s003.pdf]

Rebuttal letter

For the manuscript “Survey of clot waveform analysis of normal activated partial thromboplastin time in patients with cirrhosis and sepsis at Le Van Thinh hospital” [PONE-D-24-41352]

Response to reviewers

We sincerely appreciate the valuable time and effort the reviewers and editors have dedicated to evaluating our manuscript. Their insightful comments and constructive feedback have contributed significantly to enhancing the clarity, rigor, and overall quality of our work. We have carefully addressed all concerns and have made substantial revisions to align with the expectations for major revisions.

To ensure transparency and clarity, we have provided a detailed, point-by-point response to each comment. Each response is structured and corresponds directly to the respective reviewer’s query. Additionally, we have made significant improvements in the writing throughout the manuscript to enhance readability and better communicate the core aspects and contributions of our study.

---

1. Response to Academic Editor:

Please include the following items when submitting your revised manuscript:

• A rebuttal letter that responds to each point raised by the academic editor and reviewer(s). You should upload this letter as a separate file labeled 'Response to Reviewers'.

Authors’ response: That’s included

• A marked-up copy of your manuscript that highlights changes made to the original version. You should upload this as a separate file labeled 'Revised Manuscript with Track Changes'.

Authors’ response: That’s included

• An unmarked version of your revised paper without tracked changes. You should upload this as a separate file labeled 'Manuscript'.

Authors’ response: That’s included

2. Response to Journal requirements:

Comment-1: Please ensure that your manuscript meets PLOS ONE's style requirements, including those for file naming.

Authors' response: Thank you very much for your comments. We formatted the manuscript based on the instruction guidelines defined in the template.

Comment-2: We note that your Data Availability Statement is currently as follows: [All relevant data are within the manuscript and its Supporting Information files.]

Please confirm at this time whether or not your submission contains all raw data required to replicate the results of your study. Authors must share the "minimal data set" for their submission. PLOS defines the minimal data set to consist of the data required to replicate all study findings reported in the article, as well as related metadata and methods

Authors' response: Thank you for bringing this up. We confirm that the minimal dataset required to replicate the results of our study has been uploaded as part of the Supporting Information files accompanying the manuscript. This dataset includes:

- A table of values used to build means, standard deviations, other measures reported and graphs.

Comment-3: We note you have included a table to which you do not refer in the text of your manuscript. Please ensure that you refer to Tables 1-7 in your text; if accepted, production will need this reference to link the reader to the Table.

Authors' response: We apologize for not mentioning all the tables in the manuscript's text. We have added and referenced them in the manuscript.

Comment-4: Please review your reference list to ensure that it is complete and correct. If you have cited papers that have been retracted, please include the rationale for doing so in the manuscript text, or remove these references and replace them with relevant current references. Any changes to the reference list should be mentioned in the rebuttal letter that accompanies your revised manuscript. If you need to cite a retracted article, indicate the article's retracted status in the References list and also include a citation and full reference for the retraction notice

Authors' response: We reviewed the reference list. After that, we replaced the reference "National Guideline. National Institute for Health and Care Excellence: Guidelines. Cirrhosis in Over 16s: Assessment and Management. London: National Institute for Health and Care Excellence (NICE)" (reference No.5 in old manuscript) by "Bitto N, Liguori E, La Mura V. Coagulation, Microenvironment and Liver Fibrosis. Cells. 2018;7(8):85." (reference No.5) and "Airola C, Pallozzi M, Cerrito L, Santopaolo F, Stella L, Gasbarrini A, et al. Microvascular Thrombosis and Liver Fibrosis Progression: Mechanisms and Clinical Applications. Cells. 2023;12(13):1712." (reference No.6). Besides, we have added one reference

"Kujovich JL. Coagulopathy in liver disease: a balancing act. Hematology American Society of Hematology Education Program. 2015;2015:243-9." (reference No.9). We have carefully double-checked the reference list and confirm its completeness and accuracy. We verified that none of the cited papers have been retracted.

2. Response to Reviewer-1:

Comment-1: Line 80: The three Full stops (...) at the end of the sentence are not necessary.

Authors' response: We have removed the unnecessary ellipsis at the end of the sentence in line 80.

Comment-2: Image resolution needs to be improved

Authors' response: We have enhanced the resolution of all figures to meet the publication standards of PLOS ONE by using the PLOS-provided tool PACE. High-quality images have been uploaded in compliance with the journal's guidelines.
