# Supplementary material for: Survey of clot waveform analysis of normal activated partial thromboplastin time in patients with cirrhosis and sepsis at Le Van Thinh hospital
Source: PLoS One. 2025 Oct 24;20(10):e0334920. doi: 10.1371/journal.pone.0334920 (PMC12551873; doi:10.1371/journal.pone.0334920)
Supplement: Attachment — Submitted filename: Response to reviewers.docx [file pone.0334920.s005.docx]

**Rebuttal letter**

For the manuscript “Survey of clot waveform analysis of normal activated partial thromboplastin time in patients with cirrhosis and sepsis at Le Van Thinh hospital” [PONE-D-24-41352R2]

**Response to reviewers**

We sincerely appreciate the valuable time and effort the reviewers and editors have dedicated to evaluating our manuscript. Their insightful comments and constructive feedback have contributed significantly to enhancing the clarity, rigor, and overall quality of our work. We have carefully addressed all concerns and have made substantial revisions to align with the expectations for minor revisions.

To ensure transparency and clarity, we have provided a detailed, point-by-point response to each comment. Each response is structured and corresponds directly to the respective reviewer’s query. Additionally, we have made significant improvements in the writing throughout the manuscript to enhance readability and better communicate the core aspects and contributions of our study.

**1. Response to Journal Requirements:**

**Comment-1**: If the reviewer comments include a recommendation to cite specific previously published works, please review and evaluate these publications to determine whether they are relevant and should be cited. There is no requirement to cite these works unless the editor has indicated otherwise.

**Authors’ response:**

In line with the journal’s guidance, we reviewed the works suggested by the reviewers and cited those that are directly relevant and improve the interpretation of our findings. Specifically, we added:

- [19] Suzuki A, Suzuki N, Kanematsu T, Shinohara S, Arai N, Kikuchi R, et al. Clot waveform analysis in Clauss fibrinogen assay contributes to classification of fibrinogen disorders. Thrombosis Research. 2019;174:98-103: Discussion, paragraph 9.

- [20] Siegemund T, Scholz U, Schobess R, Siegemund A. Clot waveform analysis in patients with haemophilia A. Hamostaseologie. 2014;34 Suppl 1:S48-52: Discussion, paragraph 9.

- [21] Wong WH, Tan CW, Abdul Khalid NB, Dalimoenthe NZ, Yip C, Tantanate C, et al. Reagent Effects on the Activated Partial Thromboplastin Time Clot Waveform Analysis: A Multi-Centre Study. Diagnostics (Basel, Switzerland). 2023;13(14) : Discussion, paragraph 9.

**Comment-2:** Please review your reference list to ensure that it is complete and correct. If you have cited papers that have been retracted, please include the rationale for doing so in the manuscript text, or remove these references and replace them with relevant current references. Any changes to the reference list should be mentioned in the rebuttal letter that accompanies your revised manuscript. If you need to cite a retracted article, indicate the article’s retracted status in the References list and also include a citation and full reference for the retraction notice

**Authors’ response:**

Thank you for your insightful comment. In accordance with the journal’s guidance, we reviewed the entire reference list for completeness and accuracy and screened all citations for retraction status as of August 17, 2025. We confirm that no cited items have been retracted.

We added several current and directly relevant publications to strengthen context and interpretation, as listed below and reflected in the revised manuscript and clean reference list.

- [15]. Angeli P, Bernardi M, Villanueva C, Francoz C, Mookerjee RP, Trebicka J, et al. EASL Clinical Practice Guidelines for the management of patients with decompensated cirrhosis. Journal of Hepatology. 2018;69(2):406-60 – added to Study design, paragraph 2.

- [18]. Thanapirom K, Suksawatamnuay S, Thaimai P, Ananchuensook P, Kijrattanakul P, Angchaisuksiri P, et al. Association between Clot Waveform Analysis Parameters and the Severity of Liver Cirrhosis. Thrombosis and haemostasis. 2025 – added to Discussion, paragraph 5.

- [19]. Suzuki A, Suzuki N, Kanematsu T, Shinohara S, Arai N, Kikuchi R, et al. Clot waveform analysis in Clauss fibrinogen assay contributes to classification of fibrinogen disorders. Thrombosis Research. 2019;174:98-103 – added to Discussion, paragraph 9.

- [20]. Siegemund T, Scholz U, Schobess R, Siegemund A. Clot waveform analysis in patients with haemophilia A. Hamostaseologie. 2014;34 Suppl 1:S48-52 – added to Discussion, paragraph 9.

- [21]. Wong WH, Tan CW, Abdul Khalid NB, Dalimoenthe NZ, Yip C, Tantanate C, et al. Reagent Effects on the Activated Partial Thromboplastin Time Clot Waveform Analysis: A Multi-Centre Study. Diagnostics (Basel, Switzerland). 2023;13(14) – added to Discussion, paragraph 9.

**2. Response to Reviewer #3:**

**Comment-1**: Suggest to remove the ":" from these few lines since the referred tables or figures might not follow the line (subject to the editor's discretion) - Lines 220, 269, 283, 297.

**Authors’ response**:

We agree with the reviewer. We have removed the colon following at lines 220, 269, 283, and 297. The changes are shown in the tracked-changes version.

**Comment-2**: Minor typo error on Line 382: "which limis their accessibility" to "which limits their accessibility".

**Authors’ response**:

We agree with the reviewer. We have corrected the typo on Line 382 from “which limis their accessibility” to “which limits their accessibility.” We reviewed the manuscript for similar minor errors and corrected them; no further instances remain in the revised version.
